# Time-Dependent Behavior of Full-Scale Recycled Aggregate Concrete Beams under Long-Term Loading

**DOI:** 10.3390/ma13214862

**Published:** 2020-10-29

**Authors:** Wanlin Cao, Yibin Liu, Qiyun Qiao, Yu Feng, Shiyang Peng

**Affiliations:** College of Architecture and Civil Engineering, Beijing University of Technology, Beijing 100124, China; wlcao@bjut.edu.cn (W.C.); qiaoqiyun@bjut.edu.cn (Q.Q.); wangfeisdjz@163.com (Y.F.); guoruijie1997@163.com (S.P.)

**Keywords:** recycled aggregate concrete, full-scale beam, long-term loading, time-dependent behavior

## Abstract

Sixteen full-scale recycled aggregate concrete (RAC) beams were cast and tested to study their time-dependent behavior under long-term loading. The test parameters include the replacement rate of the recycled coarse aggregates (RCAs), the replacement rate of the recycled fine aggregates (RFAs), the concrete strength, and the stress ratio. The influence of the above variables on the deflection and stiffness is discussed. The results show that the RCAs will increase the deflection of the specimen at a low stress ratio; at a high stress ratio, the beams will experience nonlinear creep, and the deflection of the specimen will be affected by the RCAs and the stress ratio. The RFAs have a substantial influence on the time-dependent behavior of the specimen, and the deflection of the specimen with 100% replacement of recycled aggregates can increase by 30%; the strength of the concrete does not have a substantial influence on the time-dependent behavior of the specimen; and the stress ratio has an influence on the initial deflection of the specimen. A deflection calculation formula is established based on the stress–strain relationship of the beam under long-term loading and the characteristics of the RAC. The calculation results are in good agreement with the test results.

## 1. Introduction

Recycled aggregate concrete (RAC) is a kind of concrete that is made by replacing different percentages of the coarse and fine aggregates in natural aggregate concrete (NAC) with coarse and fine aggregates obtained by waste materials, such as, waste concrete, brick, tile, and rubber [1,2]. Research on and applications of RAC are an important part of the resource utilization of construction waste concrete. First, RAC is only used for nonstructural components because of its low compressive strength and the complex interface relationship of the recycled aggregates. In recent years, research and engineering applications of recycled concrete components and structures have gradually increased, which has effectively promoted the application and development of recycled concrete structures. A large number of scholars have studied many aspects of the mechanical properties of recycled concrete, including research on the macroperformance [3,4,5,6] and influencing factors [7,8,9], as well as theoretical and numerical analyses [10,11,12], and many important results have been achieved.

The time-dependent behavior is an important index of the long-term behavior of RAC. It leads to the redistribution of internal forces among different members, redistribution of the stresses in the concrete and steel of the same member section, and increases in the deformation of members under long-term loading. The deformation of RAC under long-term loading is mainly composed of instantaneous deformation under loading and continuous deformation under long-term loading. The instantaneous deformation of RAC mainly depends on the stiffness of the component, and the continuous deformation mainly includes creep and shrinkage. Geng et al. [13,14], Domingo-cabo et al. [15], Gómez-Soberon [16], Seara-Paz et al. [17], and Fathifazl et al. [18] studied the performance of RAC under long-term loading, and Chao-Qun Lye et al. [19] analyzed the strain of RAC under long-term loading. A large number of studies have shown that the regular pattern of time-dependent behavior of RAC is similar to that of NAC, but the deformation of RAC is larger than that of NAC; the more recycled aggregates that are added, the more obvious the increase is, and increases in the concrete strength can reduce the influence of the recycled aggregates to a certain extent.

Although numerous researchers have studied RAC beams, most full-scale structural studies have only focused on short-term RAC beam behavior [20,21,22,23,24]. Research has shown that the loading process of RAC beams is basically the same as that of NAC beams. The calculation can refer to ordinary concrete beams, but the influence of the recycled aggregates should be considered. In the study of the time-dependent behavior of RAC beams [25,26,27], it is generally accepted that the addition of recycled aggregates will increase the long-term deflection of RAC beams because of the large creep and shrinkage of RAC. However, up to now, only a limited number of studies have actually included full-scale structural tests, and most studies on the time-dependent behavior of RAC beams have been performed with small-scale specimens [28,29]. In fact, there have been some studies on the size effect of natural aggregate concrete [30,31,32]. The studies show that the performance of full-scale specimen is different from that of a small-scale specimen, which indicates that the size effect is a problem that cannot be ignored in engineering applications. With the application of RAC, a large number of engineering applications are based on the research on small-size RAC specimens, and the small-size specimens weakens the influence of the interface characteristics of the recycled aggregates on the working performance of the specimens to a certain extent. Therefore, it is necessary to study full-scale specimens and then apply the results to engineering to solve the size effect. Moreover, most studies have only considered single influencing factors [33,34]. For calculation of the long-term deflection of RAC beams, a large number of studies are based on a partial correction by considering some influencing factors [35,36,37,38], which cannot accurately measure the long-term deflection of recycled concrete beams. In summary, previous research on the time-dependent behavior of RAC beams has tended to consider small samples and only a few influencing factors. It is important to study the influence of different factors on the time-dependent behavior of full-scale RAC beams through experiments and to then propose a long-term deflection calculation formula for RAC beams.

Therefore, to study the time-dependent behavior of full-scale RAC beams under different influencing factors, 16 full-scale RAC beam specimens are tested under long-term loading scenarios. The parameters of the specimens include the replacement rate of the recycled coarse aggregates (RCAs), the replacement rate of the recycled fine aggregates (RFAs), the strength of the concrete, and the stress ratio. The influence of the design parameters on the time-dependent behavior of the specimens is analyzed by studying the deflection-load holding time curves and the midspan stiffness degradation of the specimens. Based on the stress–strain analysis of the beams and the characteristics of the RAC, a long-term deflection calculation formula for RAC beams is established.

## 2. Experimental Investigation

### 2.1. Test Specimens

**Figure 1:**
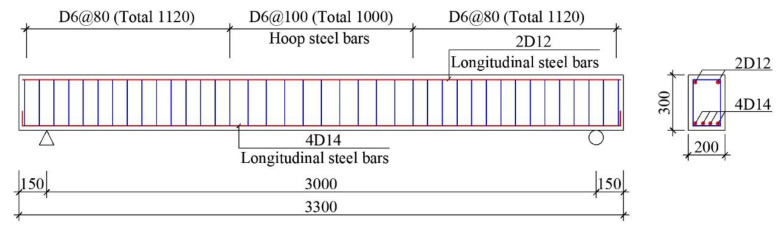
Dimensions and reinforcement of the specimen (unit: mm).

To study the influence of different factors on the time-dependent behavior of RAC beams, 16 beams were designed and manufactured in accordance with the requirements of GB 50010-2010 [39], and the dimensions and reinforcement of the specimens are shown in Figure 1. The size of the specimen was 200 mm × 300 mm × 3300 mm, and the spacing between the supports was 3000 mm. Two longitudinal bars with diameters of 12 mm were arranged at the upper end of the beam, and four longitudinal bars with diameters of 14 mm were arranged at the lower end; the reinforcement ratio of the beam was 1.4%, which is common in engineering. The diameter of the hoop reinforcement was 6 mm, and the cover depth of reinforcement was 20 mm. The number of specimens and the actual stress ratio are shown in Table 1. In Table 1, D represents RAC with a design strength of 40 MPa, which is called moderate strength concrete; G represents RAC with a design strength of 60 MPa, which is called high strength concrete; H represents a stress ratio of the specimen that is higher than 0.4, which is called a high stress ratio; L represents a stress ratio of the specimen that is less than 0.4, which is called a low stress ratio; 100/50 represents a 100% replacement rate of RCAs and a 50% replacement rate of RFAs, and the rest are similar; *M* is the actual bending moment due to loading; *M*_y_ is the yield bending moment; and *M*/*M*_y_ is the actual stress ratio. The replacement rate of RCAs is 0%, 33%, 66%, and 100%, and the replacement rate of RFAs is 0%, 50%, and 100%, which is commonly used in research.

### 2.2. Materials

#### 2.2.1. Coarse and Fine Aggregates

**Figure 2:**
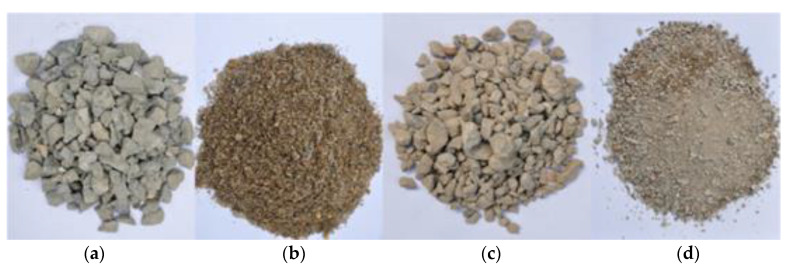
Aggregate sample. (**a**) NCAs; (**b**) NFAs; (**c**) RCAs; (**d**) RFAs.

**Figure 3:**
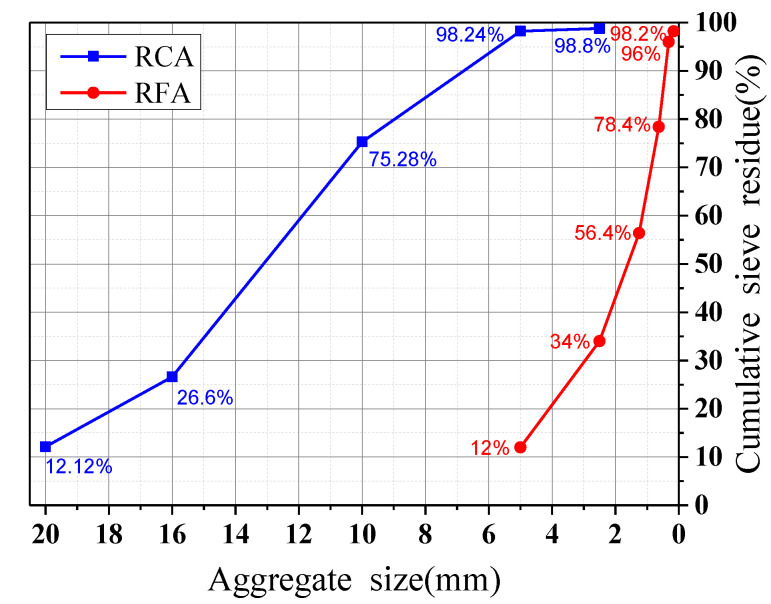
Particle size distribution of the RCAs and RFAs.

**Figure 4:**
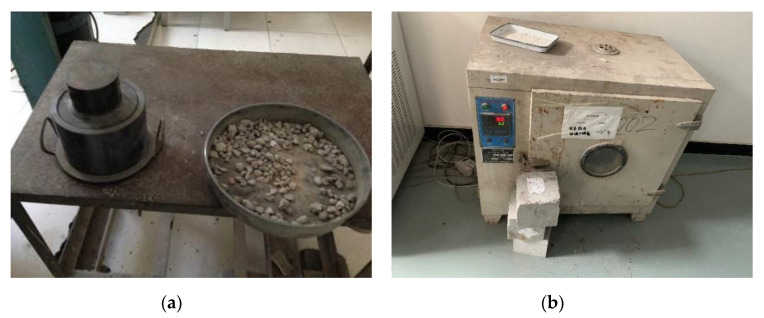
Instruments used for measuring the basic properties of the aggregates. (**a**) Crushing index; (**b**) Water absorption.

Photographs of the aggregates adopted in the experiment are shown in Figure 2. The natural coarse aggregates (NCAs) used in the test came from mountain gravels, and the natural fine aggregates (NFAs) came from river sands, with a fineness modulus of 2.6. The recycled aggregate came from a demolished concrete structure with over 20 years of service in Beijing. After sieving, aggregates with a particle size of 5–25 mm were used as recycled coarse aggregates (RCAs), and aggregates with a particle size of 0.16–5 mm were used as recycled fine aggregates (RFAs). The particle size distribution test results of the RCAs and RFAs are shown in Figure 3. According to the requirements of GB/T 14684-2011 [40], GB/T 14685-2011 [41], GB/T 25176-2010 [42], and GB/T 25177-2010 [43], the basic properties of the aggregates are shown in Table 2. Some of the instruments used for measuring the basic properties of the aggregates are shown in Figure 4. 

#### 2.2.2. Cementitious Materials

Common Portland cement (P.O.42.5) was used in the test. According to the requirements of GB 175-2007 [44], the test results were as follows: the 28 days compressive strength of the cement was 57.5 MPa, and the flexural strength was 9.2 MPa. The stability and setting time were also in line with standard GB 175-2007. To improve the durability and fluidity of the concrete, fly ash, and granulated mineral powder were used as cementitious materials instead of cement. Therefore, the water–binder ratio was used as the control variable instead of the water–cement ratio. The quality indexes of fly ash and mineral powder were in line with standards GB/T 1596-2005 [45] and GB/T 18046-2008 [46], respectively. 

#### 2.2.3. Water

Due to the high water absorption capacity of recycled aggregate, it is necessary to consider the water absorption of aggregates and add additional water on the basis of water consumption. Mixing water is daily water.

#### 2.2.4. Steel

According to the requirements of GB/T 28900-2012 [47], the mechanical properties of the longitudinal steel bars and hoop steel bars of the specimen are shown in Table 3, including the yield strength *f*_y_, ultimate strength *f*_u_, Young’s modulus *E*_s_, and elongation *δ*.

### 2.3. Mix Proportion

The design mix proportions of the concrete were in accordance with JGJ 55-2011 [48]. The mix proportions of the RAC designed with two different water–binder ratios are shown in Table 4. In Table 4, w/b is the water–binder ratio, water is the amount minus the additional water, *ρ*_c_ is the replacement rate of RCAs, *ρ*_f_ is the replacement rate of RFAs, and *f*_cu_ and *E*_c_ are the cubic compressive strength and modulus of elasticity at 28 days, respectively. 

The naphthalene superplasticizer JFL-5 with a water reducing rate of 18% was used in the test. When the water–binder ratio is 0.52, the solid content used is 5.8% to 6.5%, and the slump is controlled between 50 and 90. In the case of a water-binder ratio of 0.32, the solid content is 6.2% to 6.8%, and the slump is controlled between 70 and 120, which meets the requirements of GB/T 50080-2016 [49] and GB/T 14902-2012 [50]. 

### 2.4. Test Setup

**Figure 5:**
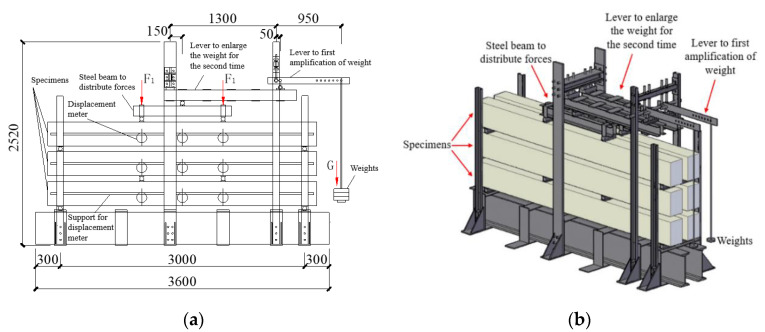
Schematic diagram of the loading device. (**a**) Plan of loading device; (**b**) stereogram of loading device (unit: mm).

In the past, the loading equipment for long-term studies was either directly loaded with heavy objects or loaded with springs or simple primary levers [25,26,27,28,29,33,34]. This leads to either the volume being too large or the device being too complicated. Therefore, aiming at solving this problem, a secondary lever loading device was designed and used for this study. This loading device is a self-balancing loading system, which is loaded by the primary lever and secondary lever for amplification, and the weight can be increased by 171 times. In the design, the lever is set in the upper space of the beam, which effectively reduces the occupied area of the arm. This loading device can load multiple concrete beams in a small test site, and the loading device is shown in Figure 5. A large amount of space can be saved by using the secondary lever loading device. A large loading force can be obtained by using a smaller loading weight compared to that of previous loading methods, and the loading is stable. The amount of weight can be adjusted at any time to obtain the required loading force. By arranging the force sensor on the upper end of the distribution beam, the exact value of the actual loading force can be obtained at any time by manual data collection. The dial indicators are arranged at the loading point of the beam span and the three-point loading point. Through manual measurement of dial indicator readings, the deflection values of the beam in the middle span and the deflection value of three stress points of simply supported bearing at both ends are obtained.

**Figure 6:**
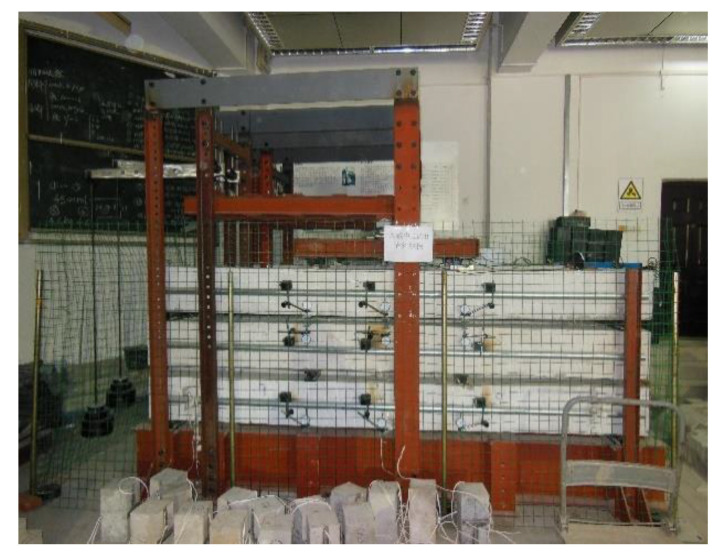
Photo of the experimental test setup.

In this study, the time-dependent behavior of sixteen beams under a 744 days long-term loading was studied in accordance with the requirements of GB/T 50152-2012 [51] by using three sets of loading devices. The age of each specimen at first loading was 28 days. Each group of loading systems can independently load the upper, middle, and lower beams. The specimens were loaded under natural conditions for Beijing, as shown in Figure 6. The average temperature was 20 °C, and the average humidity was 55%. 

## 3. Results and Discussion

### 3.1. Regular Pattern of Time-Dependent Behavior

**Figure 7:**
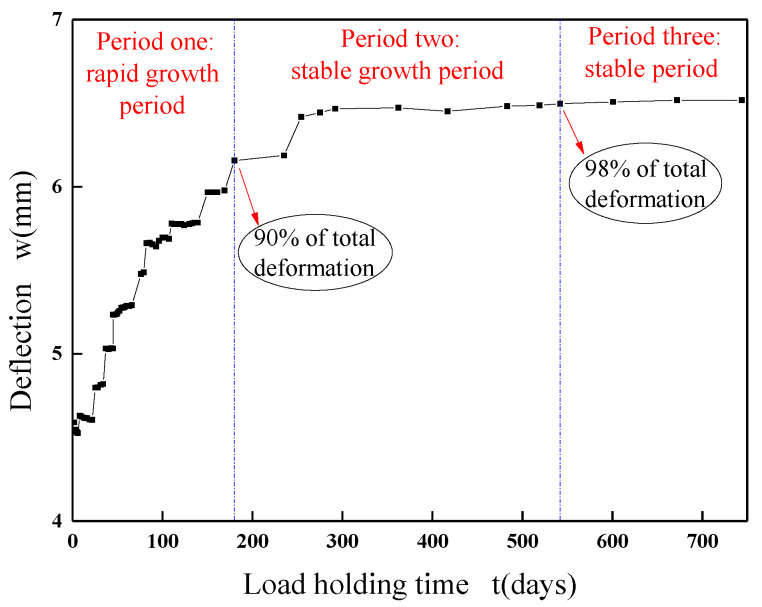
Time-dependent behavior development of D100/0-H.

The midspan deflection time history of each specimen was very similar. Taking D100/0-H as an example, its deflection-load holding time curve is shown in Figure 7.

Combined with other specimens, the time-dependent behavior process of the RAC beams can be approximately divided into three periods. The first period is the rapid growth period of time-dependent behavior, which occurs in the early stage of loading. During this period, the age of the beam is relatively young, and the deflection generated by shrinkage and creep is large, so the midspan deflection increases rapidly. During this period, the deflection of the beam can reach 90% of the total deflection in the test. The second period is the stable growth period of time-dependent behavior, which lasts from 180–540 days of loading. During this period, the shrinkage and creep increment of the RAC slow down, the deflection growth rate of the beam decreases gradually, and the deflection of the beam can be approximately 98% of the total deflection in the test. The third period is the stable period of time-dependent behavior, which takes place after 540 days of loading. During this period, the shrinkage and creep of the RAC tend to be stable, with little change. 

### 3.2. Influence of Different Factors on the Time-Dependent Behavior

The midspan deflection values of the RAC beams are listed in Table 5.

#### 3.2.1. Replacement Rate of the RCAs

**Figure 8:**
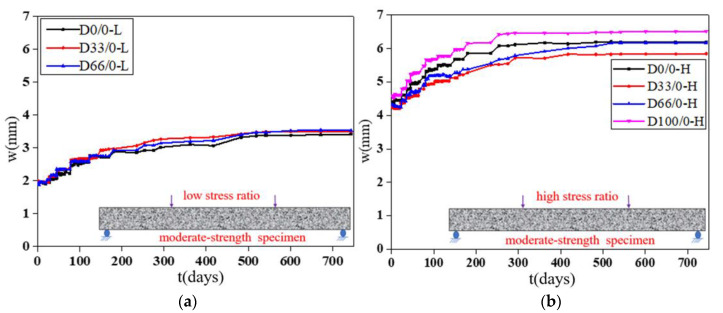
Deflection-load holding time curves of the moderate strength specimens. (**a**) Specimens under a low stress ratio; (**b**) specimens under a high stress ratio.

**Figure 9:**
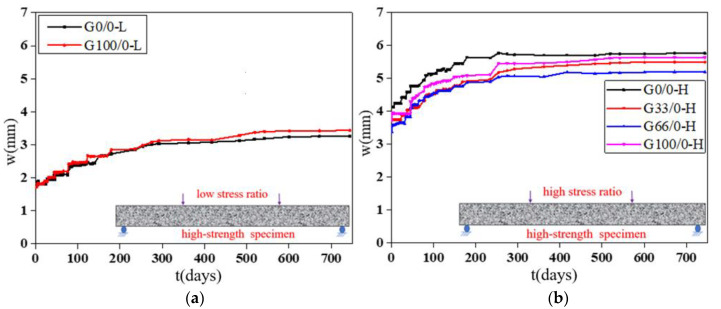
Deflection-load holding time curves of the high-strength specimens. (**a**) Specimens under a low stress ratio; (**b**) specimens under a high stress ratio.

**Figure 10:**
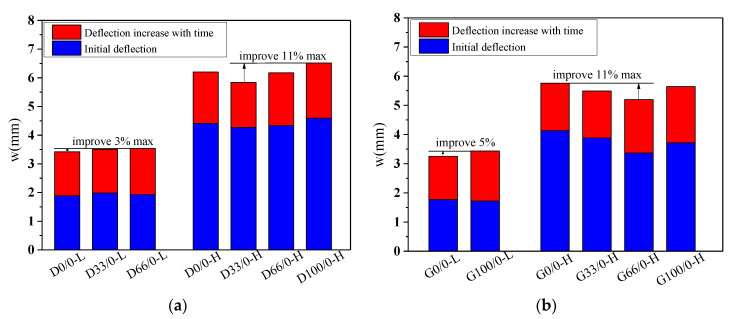
Effect of the RCAs on the ultimate deflection. (**a**) Moderate-strength specimens; (**b**) high-strength specimens.

The deflection “*w*”-load holding time “*t*” curves of the specimens with RCAs are shown in Figure 8 and Figure 9. Figure 10 shows the effect of the RCAs on the ultimate deflection in the test.

It can be seen that, at a low stress ratio, the initial deflections of the specimens with different RCA replacement ratios are almost the same, the increases over time of the deflection of the specimens with RCAs are larger than those of the NAC beams, and the maximum deflection of the beams with RCAs is 8% larger than that of the NAC beams after 744 days of load holding. This shows that the RCAs had a substantial effect on the deflection of the specimens with a low stress ratio. Because a certain amount of old cement mortar was attached to the surface of the recycled aggregates, the total amount of mortar was higher than that of the NAC; at the same time, the interface transition area of the RAC was more complex, and weak links easily formed in the interface. When RCAs are crushed, more initial microcracks are produced in the interior, which leads to large creep and shrinkage values for the RAC. However, at a high stress ratio, the conclusion is different from that of the low stress ratio study, and there is no evident effect of the RCAs on the time-dependent behavior of the specimen. When concrete is continuously loaded, it is generally considered that the strain will enter into the nonlinear stage when it exceeds 40% of the critical force, that is, the strain and the force are nonlinear, leading to a substantial increase in the deflection until damage occurs. The nonlinear deformation phenomenon is mainly caused by the microcracks on the interface between the aggregates and the cement slurry. Therefore, under a high stress ratio, the deflection of the specimen is affected by both the stress ratio and the recycled aggregates. However, due to the limited experimental data, the contribution of the two factors to the time-dependent behavior could not be determined in this study. Scholars have found that the deflection of RAC beams and the increase in RCAs have no clear trends, which also confirms the conclusions. The research performed by Adam M. Knaack [52] and Won-Chang Choi [53] shows that the deflection under long-term loading of several groups of beams with different replacement rates of recycled aggregates exhibits only slight differences for a certain load holding time.

#### 3.2.2. Replacement Rate of the RFAs

**Figure 11:**
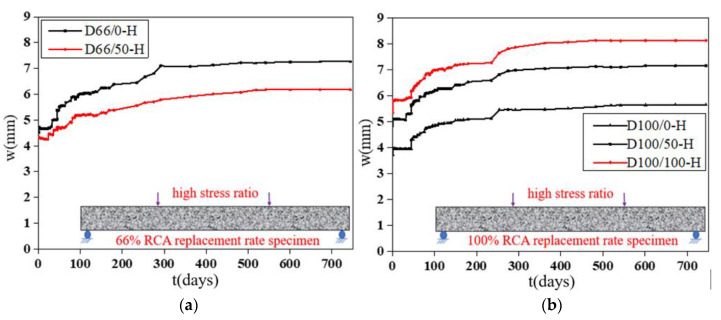
Deflection-load holding time curves of the specimens with different RFA replacement ratios. (**a**) Specimens with a 66% RCA replacement rate; (**b**) specimens with a 100% RCA replacement rate.

**Figure 12:**
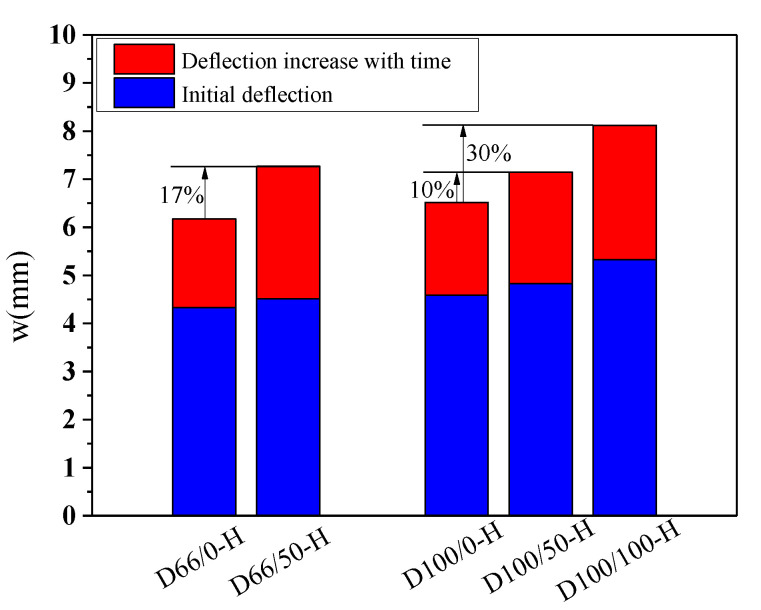
Effect of the RFAs on the ultimate deflection.

The deflection “*w*”-load holding time “*t*” curves of the specimens with RFAs are shown in Figure 11. Figure 12 shows the effect of the RFAs on the ultimate deflection in the test.

It can be seen that the effect of the RFAs on the time-dependent behavior of the specimens is very obvious. The deflection values of the specimens with RFAs are much larger than those without RFAs, and the higher the replacement rate is, the larger the deflection values are. When the coarse and fine aggregates are completely replaced, the deflection of the specimen will increase by 30%. This occurs because the RFAs are mainly composed of sand attached to hardened cement paste, hardened cement paste debris, soil, and impurities produced by aggregate crushing, which leads to worse binding capacities and stability of the RFAs. Compared with the RCAs, the RFAs will substantially reduce the time-dependent behavior of RAC, the creep and shrinkage of the concrete will increase more obviously, the composition of the RFAs is more complex, the quality is more difficult to control, and the performance of the concrete will fluctuate greatly. 

#### 3.2.3. Strength of the Concrete

By comparing the deflection-load holding time curves of the two groups with different concrete strengths in Figure 8a and Figure 9a, Figure 8b and Figure 9b, it can be seen that the influence of the concrete strength on the time-dependent behavior is relatively small, and the deflection of the specimen is mainly affected by the recycled aggregates and the stress ratio. However, the water–cement ratio, aggregate quality, and gradation not only affect the actual strength of the RAC in the preparation process but also have some influence on the time-dependent behavior. Therefore, it is recommended that the influence of the water–cement ratio, aggregate quality, and gradation on the time-dependent behavior of the RAC should be directly considered in future studies to replace the influence of the concrete strength factor.

#### 3.2.4. Stress Ratio

**Figure 13:**
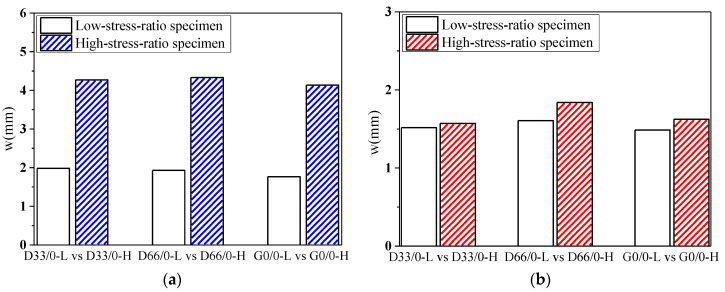
Effect of the stress ratio on the deflection of the specimens. (**a**) Initial deflection of the specimens; (**b**) deflection increases of the specimens with time.

Figure 13 shows the effect of the stress ratio on the deflection of the specimens. Compared with the low stress ratio, the initial deflection of the specimens increases greatly at a high stress ratio, and the deflection increases slightly with time. This occurs because the specimen will produce a larger instantaneous deflection under a large loading force. At the high stress ratio in this study, the nonlinear deformation of the specimen just begins, so the deflection will increase slightly with time compared to that at a low stress ratio.

### 3.3. Stiffness Analysis

Under the long-term loading, the deflection values of the specimens increase with time, and the stiffness decreases. The midspan stiffness “*B*” can be calculated by Equation (1) from the deflection “*w*”.

(1) $$B=\frac{23Fl^{3}}{648w}$$

where *l* is the distance between the two supports.

**Figure 14:**
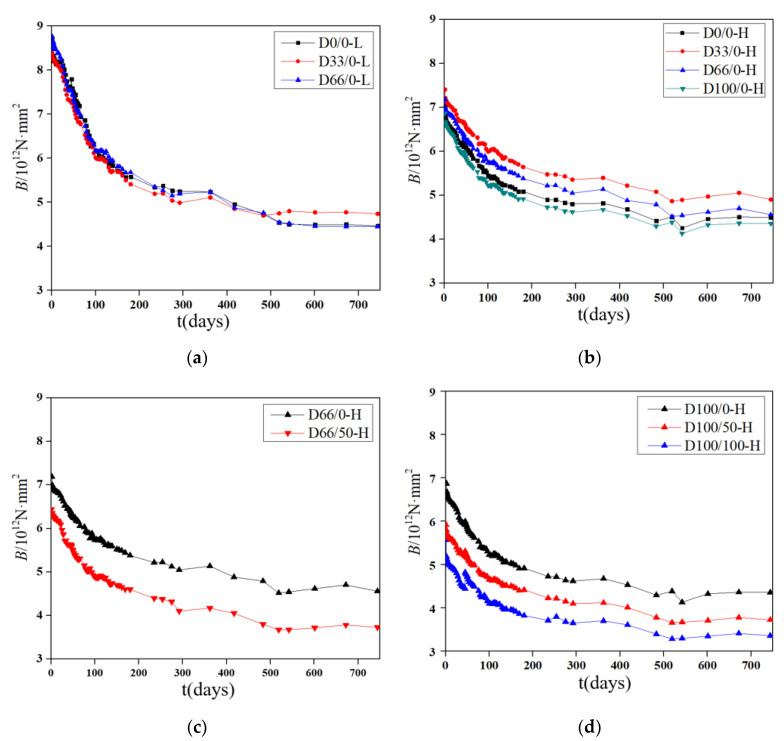
Curves of the midspan stiffness with the load holding time. (**a**) Specimens with RCAs under a low stress ratio; (**b**) specimens with RCAs under a high stress ratio; (**c**) specimens with a 66% RCA replacement rate; (**d**) specimens with a 100% RCA replacement rate.

At different stress ratios, curves the midspan stiffness “*B”* of the specimens with different RCA replacement rates with the load holding time “*t”* are shown in Figure 14a,b. It can be seen that the change trends of the stiffnesses of the specimens under different RCA replacement rates are similar, and there is no obvious difference in the stiffnesses of the specimens at a low stress ratio; at a high stress ratio, the regularity of the stiffness is the same as its compressive strength, and there is no obvious relationship with the RCA replacement rate. This indicates that the midspan stiffnesses of the specimens are not substantially related to the RCA replacement rate.

At different stress ratios, the midspan stiffnesses “*B*” of the specimens with different RFA replacement rates with load holding time “*t*” are shown in Figure 14c,d. It can be seen that the increase in the RFA replacement rate will substantially reduce the midspan stiffness of the specimens when the RCA replacement rate is fixed.

## 4. Theoretical Formula for the Deflection Calculation

### 4.1. Calculation Formula Based on Long-Term Deformation Theory of Concrete

The total deflection of a concrete beam under long-term loading can be divided into three parts: the initial deflection caused by loading, the deflection increment caused by concrete creep, and concrete shrinkage.

**Figure 15:**
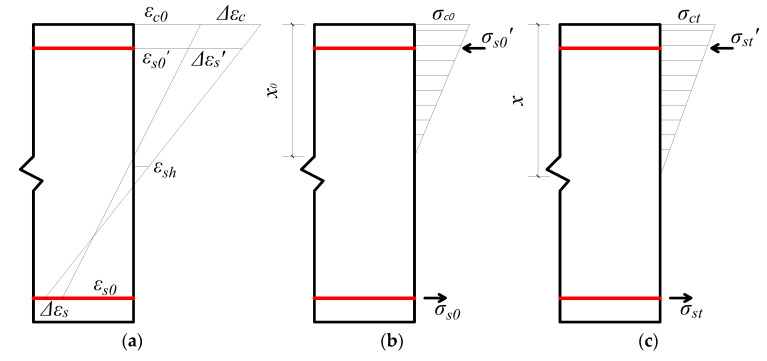
Stress–strain distribution relationship of the beam section. (**a**) Strain of the beam section at *t_0_* and *t*; (**b**) stress of the beam section at *t_0_*; (**c**) stress of the beam section at *t.*

Under the effect of long-term loading, the stress–strain distribution of the concrete beam section is shown in Figure 15.

*t*_0_ is defined as the initial moment when the beam is loaded to a stable long-term load, and *t* is any time point in the long-term loading process. In the process of loading, the tensile and compression bars endure a fixed bending moment. Due to the effect of shrinkage and creep, the concrete strain in the compression zone increases with time, but the reinforcement will not shrink or creep. Therefore, the deformation of the concrete in the compression zone will be confined by the compression reinforcement. The compressive stress in the concrete will continue to transfer to the reinforcement with time, and the internal force will continue to redistribute. Due to the fixed bending moment, to maintain the stress balance of the section, the height of the compression area of the concrete will continue to increase. Therefore, the shrinkage and creep of the concrete are the main cause of the increase in the deformation of the concrete beam with time.

In the long-term deflection calculation of concrete beams, the following basic assumptions are adopted [54,55]:

(1) Under the effect of short-term loading, the concrete in the tension area cracks, and the stress of the concrete in the compression area is not considered.
(2) Under the effect of long-term loading, the plane section assumption is still valid.
(3) Under long-term loading, the reinforcement and concrete work together without relative slip.
(4) Under long-term loading, the stress of the tensile steel bars changes only slightly and can be ignored.
(5) The calculation is performed by the age-adjusted effective modulus method.

In the time period from *t*_0_ to *t*, as the increment of the internal force and moment is 0, Equations (2) and (3) can be obtained.

(2) $$\Delta N_{c}(t,t_{0})+\Delta N_{s}^{\prime }(t,t_{0})+\Delta N_{s}(t,t_{0})=0$$

(3) $$N_{c}(t)(h_{0}-\frac{x}{3})-N_{c}(t_{0})(h_{0}-\frac{x_{0}}{3})+\Delta N_{s}^{\prime }(t,t_{0})(h_{0}-a_{s}^{\prime })=0$$

where $\Delta N_{c}(t,t_{0})$, $\Delta N_{s}(t,t_{0})$, and $\Delta N_{s}^{\prime }(t,t_{0})$ are the increment of the resultant force of the concrete compressive stress, the compression reinforcement, and the tensile reinforcement from *t*_0_ to *t*, respectively; $N_{c}(t)$ and $N_{c}(t_{0})$ are the resultant compressive stresses of the concrete at *t* and *t*_0_, respectively; *h*_0_ is the effective height of the beam section; *x* and *x_0_* are the heights of the compression zone of the beam section at *t* and *t*_0_, respectively; and $a_{s}^{\prime }$ is the distance from the resultant point of the compression reinforcement to the edge of the compression zone of the concrete.

Equations (4) and (5) can be obtained due to the plane section assumption and the age-adjusted effective modulus method.

(4) $$\Delta \psi =\frac{\Delta \varepsilon_{c}}{d}=\frac{\Delta \varepsilon_{s}^{\prime }}{d-a_{s}^{\prime }}=\frac{\Delta \varepsilon_{s}}{h_{0}-d}$$

(5) $$E_{c}(t,t_{0})=\frac{E_{c0}}{\left[1+\chi (t,t_{0})\phi (t,t_{0})\right]}$$

where $\Delta \varepsilon_{c}$, $\Delta \varepsilon_{s}$, and $\Delta \varepsilon_{s}^{\prime }$ are the strain adjustment values of the concrete at the edge of the compression zone, the tensile reinforcement, and the compression reinforcement of the beam from *t*_0_ to *t*, respectively; $E(t,t_{0})$ is the effective elastic modulus of the concrete adjusted by age at *t*; $E_{c0}$ is the modulus of elasticity of the concrete at *t*_0_; $\chi (t,t_{0})$ is the age adjusted coefficient, which can be simplified as χ; and $\phi (t,t_{0})$ is the creep coefficient of the concrete from *t*_0_ to *t*, where the creep coefficient is the ratio of the creep strain $\varepsilon_{cc}$ to the initial elastic strain under loading $\varepsilon_{c0}$, which can be simplified as ϕ.

From *t*_0_ to *t*, the adjustment of the concrete compressive strain at the edge of the compression zone of the beam section $\Delta \varepsilon_{c}$ can be expressed by Equation (6).

(6) $$\Delta \varepsilon_{c}=\varepsilon_{c0}\cdot \phi +\frac{\Delta \sigma_{c}}{E_{c}(t,t_{0})}+\varepsilon_{sh}(t,t_{0})$$

where $\Delta \sigma_{c}$ is the adjustment of the compressive stress of the concrete from *t*_0_ to *t*, and $\varepsilon_{sh}(t,t_{0})$ is the shrinkage strain of the concrete from *t*_0_ to *t*, which can be simplified to $\varepsilon_{sh}$.

The increment of the sectional curvature can be expressed by Equation (7).

(7) $$\Delta \psi =\frac{\Delta \varepsilon_{c}}{d}=\frac{\varepsilon_{c0}\left[\phi -1-\chi \phi +\frac{x_{0}}{x}(\chi \phi +1)\right]+\varepsilon_{sh}}{h_{0}\left[1+\frac{2\alpha_{E}\rho^{\prime }\left(1-a_{s}^{\prime }/h_{0}\right)}{x/h_{0}}(\chi \phi +1)\right]}$$

It can be seen that the parameters of the formula are relatively complex. To simplify the calculation, some parameters are simplified on the basis of meeting the following accuracy requirements [56]:

(1) $a_{s}^{\prime }/h_{0}$ should be taken as 0.1, under the condition of meeting the structural requirements of beam section reinforcements.
(2) χ is usually taken as 0.8.
(3) $\frac{x}{h_{0}}$ can be regarded as $\frac{x}{x_{0}}\cdot \frac{x_{0}}{h_{0}}$; the approximate values are 1.25 and 0.3, respectively.

The simplified expression of the additional curvature Δψ can be expressed by Equation (8).

(8) $$\Delta \psi =\Delta \psi_{c}+\Delta \psi_{s}=\psi_{0}\frac{x_{0}}{h_{0}}\frac{0.84\phi -0.2}{1+12.5\alpha_{E}\rho^{\prime }}+\frac{\varepsilon_{sh}}{h_{0}}\frac{1}{1+12.5\alpha_{E}\rho^{\prime }}$$

where $\Delta \psi_{c}$ is the curvature caused by creep, and $\Delta \psi_{s}$ is the curvature caused by shrinkage.

The deflection of the beam caused by creep can be related to the creep curvature through the virtual work principle, and the curvature caused by shrinkage is constant along the longitudinal direction of the beam. The calculation formula of the creep and shrinkage deflection can be expressed by Equations (9) and (10).

(9) $$\Delta f_{cr}=\int_{0}^{l}\Delta \psi_{c}(x)\bar{M}(x)dx$$

(10) $$\Delta f_{sh}=\frac{l^{2}}{8}\Delta \psi_{s}$$

The midspan deflection of the concrete beam under long-term loading can be expressed by Equation (11).

(11) $$f=\Delta f_{cr}+\Delta f_{sh}=f_{0}+\frac{f_{0}x_{0}}{h_{0}}\frac{0.84\phi -0.2}{1+12.5\alpha_{E}\rho^{\prime }}+\frac{l^{2}\varepsilon_{sh}}{8h_{0}}\frac{1}{1+12.5\alpha_{E}\rho^{\prime }}$$

The simplified formula parameters are relatively simple, and the midspan deflection of the beam under long-term loading can be calculated by determining the creep coefficient ϕ and shrinkage strain $\varepsilon_{sh}$.

### 4.2. Calculation Method of the Initial Deflection

The deflection of the beams under loading is calculated by Equation (12).

(12) $$f_{0}=\frac{Fa}{24B_{s}}(3l^{2}-4a^{2})\pm \frac{5ql^{4}}{384B_{s}}$$

where $B_{s}$ is the short-term stiffness of the concrete beam, a is the distance from the support to the loading point, and l is the distance between the two supports.

The short-term stiffness of the beams can be calculated by the stiffness analysis method. The calculation formula of the stiffness based on the analytical method in GB50010-2010 [39] is given in Equation (13).

(13) $$B_{S}=\frac{E_{S}A_{S}h_{0}{}^{2}}{1.15\psi +0.2+6\alpha_{E}\rho }$$

where $E_{S}$ is the elastic modulus of the reinforcement; $A_{S}$ is the total section area of the tensile reinforcement; ψ is the nonuniformity coefficient of the longitudinal tensile reinforcement, $\psi =1.1-0.65f_{t}/(\rho_{te}\sigma_{s})$; $f_{t}$ and $f_{\mathrm{cu}}$ are the axial tensile strength and compressive strength of the concrete, respectively; *α_c2_* is the reduction coefficient considering the brittleness of the concrete, with values ranging from 0.87 to 1; *σ_s_* is the steel stress at the crack section, $\sigma_{s}=M/\left(A_{S}\eta h_{0}\right)$; η is the internal force arm coefficient of the cracked section; *M* is the bending moment in the middle span of the beam; $\alpha_{E}$ is the ratio of the elastic modulus of the reinforcement to that of the concrete; *ρ* is the ratio of the reinforcement; and $\rho_{te}$ is the reinforcement ratio of the longitudinal tensile steel bars calculated by the effective tensile concrete section area, $\rho_{te}=\frac{A_{S}}{0.5bh}$.

The comparison between the deflection calculated by the formula and the measured value is shown in Table 6 and Table 7.

Table 6 shows that for the specimens without RFAs, the average ratio of the initial deflection calculated by the stiffness analysis method is 1.109, the standard deviation is 0.048, and the coefficient of variation is 0.043. This shows that the analytical method of calculating the stiffness can reasonably calculate the initial deflection of the beam and is conservative. Table 7 shows that the initial deflection of the specimens with RFAs calculated by the stiffness analysis method is often smaller than the measured value. This shows that the analytical method of calculating the stiffness cannot effectively fit the initial deflection of beams with RFAs. Considering the influence of RFAs on the mechanical behavior of concrete, the nonuniformity coefficient ψ of the longitudinal tensile reinforcement is multiplied by the correction coefficient α. For specimens with RCAs, α is taken as 1.0; for specimens with RFAs or with both RCAs and RFAs, α is taken as 1.2. The results obtained by using the improved analytical method of calculating the stiffness are more conservative than the results calculated by other methods.

### 4.3. Calculation Method of the Deflection Increment

In the formula, ϕ and $\varepsilon_{sh}$ are generally calculated by the shrinkage and creep prediction model of concrete. The common concrete prediction models of shrinkage and creep are the CEB-FIP2010 model [57] proposed by the European Concrete Association, the GL2000 model [58], and the ACI209R-92 model [59] proposed and recommended by the American Concrete Association. However, by analyzing the above models, the following observations can be made:

(1) The prediction models are for NAC, so the influence of recycled aggregates on the concrete shrinkage and creep is not considered.
(2) The model generally aims at specimens with stress ratios of less than 0.4, and the effect of a high stress ratio is not considered.

Therefore, based on the test data, the shrinkage and creep prediction model of RAC can be obtained by modifying the RAC characteristics.

**Figure 16:**
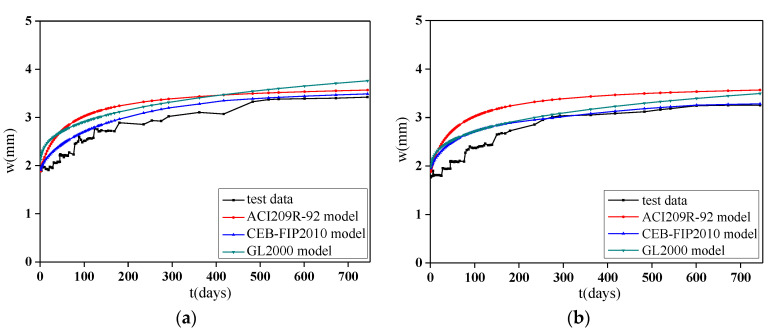
Comparison between the calculated values and the test values of each model for NAC beams under a low stress ratio. (**a**) Specimen D0/0-L; (**b**) Specimen G0/0-L.

The deflection-load holding time curves of the NAC beams under low stress ratios obtained by the above three models are shown in Figure 16. A comparison of the three curves reveals that the regular development pattern of the three models is similar to that of the experiment. The CEB-FIP2010 model is in good agreement with the test data and can reserve a certain amount of conservatism most of the time. Therefore, this model is selected as the basic model for the deflection increment calculation of RAC beams.

Compared with NAC beams, the larger self-shrinkage of the RAC is the main reason for the larger midspan deflection of the RAC beam. Based on this result, a correction coefficient is applied to the shrinkage strain of the prediction model. At a high stress ratio, the RCA and stress ratio will jointly affect the development of the deflection of the RAC beams. However, this study produced less data for high stress ratios, and the stress was more concentrated than that of other studies. Therefore, the influencing factors of the RCAs and RFAs on creep behavior are proposed, and the influencing factors of the RCAs on the creep are appropriately enlarged to ensure that the calculated deflection values of the beams under different stress ratios have a certain conservatism.

Based on the fitting results of the test data and considering the characteristics of RAC, new influence factors are proposed based on the shrinkage strain influence coefficient $\varepsilon_{cso}$:$\varepsilon_{RCA}$, which is based on the influence of the RCAs and magnified appropriately, and $\varepsilon_{RFA}$, which is based on the influence of the RFAs. The values are

$$\begin{array}{cc} \varepsilon_{RCA}=\{\begin{array}{c} \begin{array}{l} 1(\alpha \leq 30\%)\\ 1.25(30\%\text{<}\alpha \leq 70\%) \end{array}\\ \;1.4(70\%\text{<}\alpha \leq 100\%)\; \end{array} & \varepsilon_{RFA}=\{\begin{array}{c} 1.5(\alpha \leq 50\%)\\ 1.8(\alpha >50\%) \end{array} \end{array}$$

**Figure 17:**
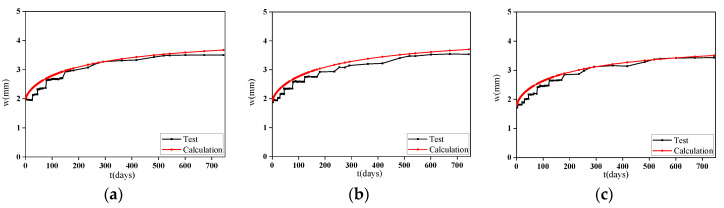

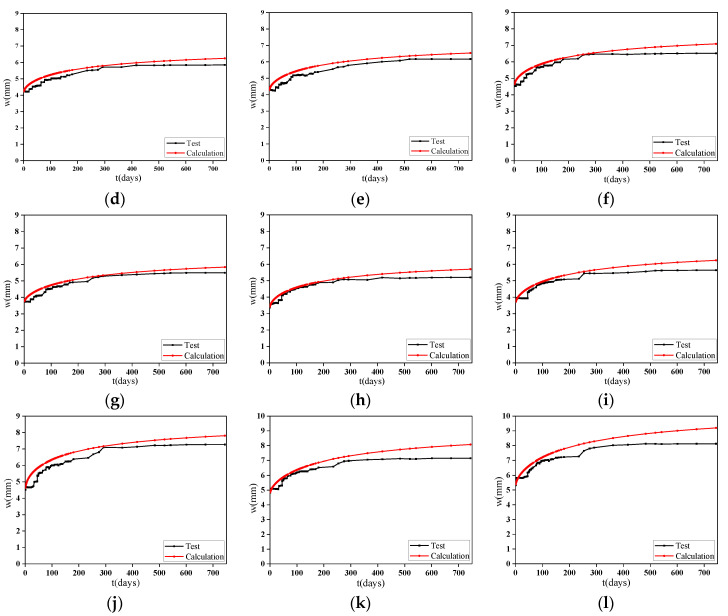
Comparison between the calculated values and the measured values of the RAC beams. (**a**) D33/0-L; (**b**) D66/0-L; (**c**) G100/0-L; (**d**) D33/0-H; (**e**) D66/0-H; (**f**) D100/0-H; (**g**) G33/0-H; (**h**) G66/0-H; (**i**)G100/0-H; (**j**)D66/50-H; (**k**) D100/50-H; (**l**) D100/100-H.

**Figure 18:**
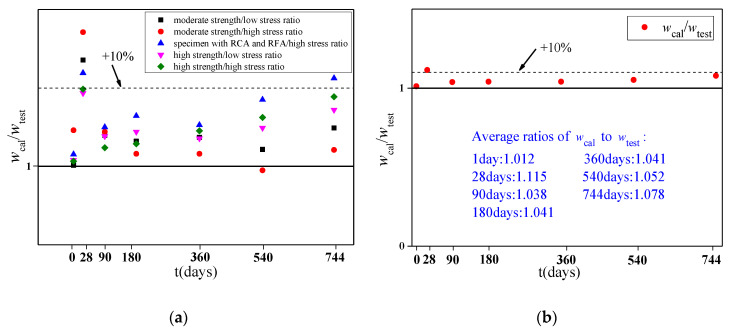
Comparisons between the test results and the calculated results. (**a**) Ratios of the different types of specimens; (**b**) average ratios of the specimens.

Based on the coefficient $\varepsilon_{RCA}$ and $\varepsilon_{RFA}$, the deflection curve of the specimen is calculated by Equations (2)–(13). Comparisons between the calculated curves and the measured deflection-load holding time curves of the RAC beams obtained by adjusting the model are shown in Figure 17. Some time periods are selected to compare the calculated results with the measured values to study their errors, including 1, 28, 90, 180, 360, 540, and 744 days. The ratio of the calculated deflection to the measured deflection of the specimens at different time periods is shown in Table 8 and Figure 18.

It can be seen from the results that the calculation values obtained by using the prediction model of RAC modified by the CEB-FIP2010 model are in good agreement with the test data. However, because the deformation of the specimen in the early period is easily affected by external factors and the development of deflection is prone to generate fluctuations, the formula is calculated under ideal conditions without considering the above factors, so the deflection calculated at a load holding time of 28 days is relatively large, with a large measured error. In the later period of loading, the convergence of the calculation model is worse than that of the measurement, especially for the specimens with RCAs and RFAs. Considering the small number of specimens, this method can meet the calculation accuracy, but it still needs to be verified with more test data.

## 5. Conclusions

In this study, the time-dependent behavior of full-scale RAC beams under the influence of different replacement ratios of RCAs and RFAs, different concrete strengths, and different load holding ratios was studied. The following conclusions were drawn:

(1) The regular time-dependent behavior pattern of full-scale RAC beams is basically the same as that of NAC beams, which is fast in the early stage and slow in the later stage until it tends to stabilize.
(2) The effect of the replacement ratio of RCAs on increases in the deflection of the beams with time is more obvious under a low stress ratio than under a high stress ratio. At a high stress ratio, the development of deflection will be affected by the RCAs and the actual stress ratio together. RFAs will obviously increase the deflection of the beam.
(3) Based on the stress–strain relationship of concrete and the characteristics of RAC, the deflection calculation formula of RAC beams under long-term loading is proposed, and the formula is optimized and verified according to the test results of full-scale specimens. The calculated values are in good agreement with the actual values.

## Figures and Tables

**Table 1:** Number of specimens and stress ratio.

Specimen	Replacement Rate of RCA/%	Replacement Rate of RFA/%	*M*/kN·m	*M*_y_/kN·m	*M/M*_y_
D0/0-L	0	0	17.48	66.5	0.26
D33/0-L	33	0	19.09	67.5	0.28
D66/0-L	66	0	19.09	57.5	0.33
D0/0-H	0	0	30.86	66.5	0.46
D33/0-H	33	0	32.46	67.5	0.48
D66/0-H	66	0	30.86	57.5	0.54
D100/0-H	100	0	32.46	61.0	0.53
D66/50-H	66	50	32.46	53.0	0.61
D100/50-H	100	50	30.86	57.5	0.54
D100/100-H	100	100	30.86	52.5	0.59
G0/0-L	0	0	22.43	77.8	0.29
G100/0-L	100	0	22.43	77.6	0.29
G0/0-H	0	0	35.81	77.8	0.46
G33/0-H	33	0	35.81	76.2	0.47
G66/0-H	66	0	35.81	76.1	0.47
G100/0-H	100	0	35.81	77.6	0.46

**Table 2:** Basic properties of the aggregates

Aggregate	Continuous Gradation (mm)	Stacking Density (kg·m^−3^)	Apparent Density (kg·m^−3^)	Crushing Value (%)	Water Absorption Ratio (%)	Sediment Percentage (%)
NCA	5–25	1580.21	2760.23	9.70	0.23	0.40
NFA	0.16–5	1560.37	2670.02	7.32	0.46	1.50
RCA	5–25	1252.80	2575.49	13.10	4.70	2.25
RFA	0.16–5	1307.46	2455.02	17.00	11.32	3.50

**Table 3:** Mechanical properties of the steel bars

Steel Type	Diameter of the Steel Bars *D* (mm)	*E*s (×10^5^ MPa)	*f*_y_ (MPa)	*f*_u_ (MPa)	*δ*(%)
Hoop steel bars	6	2.16	379	540	20.3
Longitudinal steel bars	12	1.98	421	596	25.8
	14	2.04	441	600	26.7

**Table 4:** Mix proportion of the concrete.

*w/b*	*ρ*_c_ (%)	*ρ*_f_ (%)	Coarse Aggregate (kg·m^3^)		Fine Aggregate (kg·m^3^)		Cementitious Material (kg·m^3^)			Water(kg)	*f*_cu_ (MPa)	*E*_c_ (×10 MPa)
			NCA	RCA	NFA	RFA	Cement	Fly Ash	Mineral Powder			
0.52	0	0	910	0	910	0	227	75	75	195	43.9	3.65
	33	0	610	300	910	0	227	75	75	195	40.4	3.61
	66	0	310	600	910	0	227	75	75	195	40.0	3.50
	100	0	0	820	1000	0	227	75	75	195	39.4	3.24
	66	50	280	540	500	500	227	75	75	195	38.2	3.51
	100	50	0	820	500	500	227	75	75	195	40.3	3.38
	100	100	0	730	0	1090	227	75	75	195	34.3	3.06
0.32	0	0	928	0	757	0	435	52	52	174	63.9	3.83
	33	0	622	306	757	0	435	52	52	174	57.1	3.63
	66	0	316	612	757	0	435	52	52	174	57.9	3.67
	100	0	0	928	757	0	435	52	52	174	56.6	3.55

**Table 5:** Tested deflection values of the specimens.

Specimen	*f*_cu_ (MPa)	Initial Deflection (mm)	Deflection Increase with 365d (mm)	Deflection Increase with 744d (mm)	Total Deflection (mm)
D0/0-L	35.9	1.883	1.222	1.537	3.420
D33/0-L	46.4	1.983	1.329	1.518	3.501
D66/0-L	40.0	1.930	1.272	1.607	3.537
D0/0-H	35.9	4.411	1.763	1.788	6.199
D33/0-H	46.4	4.269	1.445	1.570	5.839
D66/0-H	40.0	4.333	1.582	1.840	6.173
D100/0-H	41.4	4.590	1.882	1.927	6.517
D66/50-H	38.2	4.512	2.563	2.753	7.265
D100/50-H	41.3	4.826	2.218	2.318	7.144
D100/100-H	34.3	5.324	2.695	2.795	8.119
G0/0-L	63.9	1.767	1.287	1.487	3.254
G100/0-L	56.6	1.721	1.438	1.714	3.435
G0/0-H	63.9	4.136	1.573	1.623	5.759
G33/0-H	57.1	3.885	1.468	1.608	5.493
G66/0-H	57.9	3.368	1.68	1.830	5.198
G100/0-H	56.6	3.719	1.748	1.923	5.642

**Table 6:** Calculated initial deflection of the specimens without RFAs by the analytical method of calculating the stiffness.

Specimen	Initial Deflection (mm)	Analytical Method of Calculating the Stiffness	
		Calculated Value of the Initial Deflection (mm)	Ratio of the Calculated Value to the Measured Value
D0/0-L	1.883	2.092	1.111
D33/0-L	2.004	2.182	1.089
D66/0-L	1.930	2.235	1.158
D0/0-H	4.411	4.759	1.079
D33/0-H	4.269	4.806	1.126
D66/0-H	4.333	4.430	1.022
D100/0-H	4.590	4.919	1.072
G0/0-L	1.767	1.919	1.086
G100/0-L	1.721	2.058	1.195
G0/0-H	4.136	3.659	0.885
G33/0-H	3.885	4.158	1.070
G66/0-H	3.368	3.886	1.153
G100/0-H	3.719	4.266	1.147

**Table 7:** Calculated initial deflection of the specimens with RFAs by the improved analytical method for calculating the stiffness.

Specimen	Initial Deflection (mm)	Analytical Method of Calculating The Stiffness		Improved Analytical Method for Calculating the Stiffness	
		Calculated Value of the Initial Deflection (mm)	Ratio of the Calculated Value to The Measured Value	Calculated Value of the Initial Deflection (mm)	Ratio of the Calculated Value to the Measured Value
D66/50-H	4.512	4.510	0.999	5.059	1.121
D100/50-H	4.826	4.598	0.953	5.142	1.065
D100/100-H	5.324	4.813	0.904	5.467	1.027

**Table 8:** Ratio of the calculated deflection to the measured deflection of the specimens in different time periods.

Specimen	Load Holding Time (days)						
	1	28	90	180	360	540	744
D33/0-L	1.002	1.120	1.035	1.025	1.017	1.016	1.050
D66/0-L	1.001	1.151	1.044	1.039	1.057	1.027	1.048
G100/0-L	1.046	1.171	1.044	1.016	1.016	0.995	1.021
D66/50-H	1.028	1.184	1.078	1.065	1.035	1.053	1.074
D100/50-H	1.005	1.097	1.027	1.051	1.062	1.103	1.13
D100/100-H	1.013	1.076	1.045	1.077	1.061	1.099	1.133
D33/0-H	0.996	1.084	1.052	1.050	1.034	1.047	1.068
D66/0-H	1.008	1.099	1.037	1.071	1.042	1.035	1.060
D100/0-H	1.012	1.097	1.026	1.011	1.031	1.065	1.088
G33/0-H	1.003	1.089	1.035	1.029	1.020	1.037	1.063
G66/0-H	1.015	1.105	1.015	1.009	1.055	1.073	1.096
G100/0-H	1.002	1.101	1.021	1.048	1.061	1.077	1.107
Average	1.012	1.115	1.038	1.041	1.041	1.052	1.078
